# On the Design of Dual-Polarised Linear Antenna Arrays with Enhanced Port-to-Port Isolation

**DOI:** 10.3390/s20216105

**Published:** 2020-10-27

**Authors:** Dariusz Wójcik, Maciej Surma, Artur Noga, Mirosław Magnuski

**Affiliations:** Department of Electronics, Electrical Engineering and Microelectronics, Silesian University of Technology, Akademicka 16, 44-100 Gliwice, Poland; maciej.surma@polsl.pl (M.S.); artur.noga@polsl.pl (A.N.); miroslaw.magnuski@polsl.pl (M.M.)

**Keywords:** antenna arrays, dual polarisation, microstrip antennas, isolation

## Abstract

The paper describes the process of designing a dual-polarised linear antenna array with enhanced port-to-port isolation, with the example of a four-element array with isolation better than 60 dB for the U-NII 5.15–5.925 GHz band. As a single antenna, dual-polarised electromagnetically coupled microstrip antennas were used with port-to-port isolation not exceeding 25 dB. A significant improvement in the isolation of the array was achieved thanks to the application of a dedicated feeding network. On the basis of theoretical analysis, a mathematical model describing port-to-port isolation was developed. Circuit and full-wave simulations were carried out to show the influence of electromagnetic couplings between antennas and/or the microstrip lines of the feeding network and the selection of phase shifters/power dividers on the isolation. The fabricated prototype is characterised by a gain of about 14 dBi, polarisation purity of at least −27 dB within the main lobe and close to expectations isolation better than 57 dB within the whole operational band.

## 1. Introduction

Dual-polarisation antennas with high port-to-port isolation have wide applications in contemporary radiocommunication systems in order to increase their performance in variable propagation conditions. In case of microwave radar sensors, the sensitivity strongly depends on isolation between the RX input and TX output [1,2,3]. In 2 × 2 MIMO systems, the throughput could be doubled by means of simultaneous emission using two orthogonal polarisations [4]. In SCFD (single-channel full duplex) or STAR (simultaneous transmit and receive) systems orthogonal polarisations are utilised in full-duplex radio links working on a single frequency channel [5,6,7]. Operation of the SCFD and STAR systems requires suppression of the signal crosstalk between the transmitter output and input of the receiver at a level of 90–120 dB [5,7] with at least 60 dB realised in analog path in order to prevent the receiver from experiencing saturation effects [7]. Application of the dual-polarisation antennas with high port-to port isolation can fully or largely fulfil the expressed requirements.

Designing dual-polarised microstrip antenna arrays with increased port-to-port isolation is not a simple task, considering the fact that typical isolation of a simple dual-polarised microstrip antenna does not exceed 25 dB. Even if complex feeding methods such as a differential-driven antenna [8,9], hybrid-fed techniques [10] or aperture-coupled with specific slot shapes are taken in consideration, the maximum level of isolation will attain 40 dB [11,12]. For this reason, increasing the port-to-port isolation of microstrip antenna arrays could be achieved by applying dedicated feeding networks that utilise compensation of the crosstalk between the array ports by means of the pairwise anti-phased feeding technique where couples of radiators which are the image mirror one of another are fed by currents shifted by 180${}^{{}^{\circ}}$ in the phase [13,14,15,16,17,18]. The application of this technique contributes to an increase in isolation to over 50 dB [16,17,18] and polarisation purity to more than 36 dB [19]. In the case of multi-element uniform antenna arrays, applying parallel feeding with multiple and even power division further improvement of the port-to-port isolation could be obtained by successive mirror imaging and feeding with 180${}^{{}^{\circ}}$ phase-shifted currents of the entire radiator groups. Either ordinary T-junctions with half-wave line sections or more sophisticated networks such as directional couplers [20], baluns [21,22] and broadband phase shifters [23] could be used as power dividers and phase shifters. However, the application of the latter has a strong influence on the dimensions and complication of the feeding network (FN).

Practically achievable isolation is significantly influenced by electromagnetic couplings between antennas and/or microstrip lines of the FN. This is observed especially when the radiators and FN are located on the same layer of printed circuit board (PCB). In such a case, even after the optimisation process, it is difficult to obtain isolation better than 50 dB [13,14,24]. The achievement of higher isolation requires the use of multilayer PCBs in which radiators and FN are separated by means of ground planes [9,25,26,27]. Another important aspect of the dual-polarised antennas that affects isolation is the operational frequency band. For instance, it is much easier to obtain isolation better than 60 dB in a narrow band [8,28], while wide-band antennas usually give lower values [29,30].

In the literature, little attention is paid to linear antenna arrays which, due to their wide radiation pattern in the horizontal plane, could be used as base station antennas. Antenna arrays proposed by other authors do not offer sufficient isolation [15,23,31,32,33], or, despite satisfactory isolation, offer an operational frequency band that is too narrow [25]. This paper describes the design process of the dual-polarisation linear antenna arrays using a four-element antenna array for U-NII 5.15–5.925 GHz frequency band as an example. In comparison to earlier works by the same authors [13,14], this paper contains a detailed description of the array design: a presentation of the theoretical model for port-to-port isolation, a discussion of the influence of mechanical symmetry and couplings between the radiators and/or FN on port-to-port isolation and a description of the method of selection of the feeding network elements performed by circuit and full-wave simulations. In practical realisations of the antenna arrays described in this paper, electromagnetically coupled microstrip antennas (ECMSA) are utilised as a single radiator [34]. Full-wave and circuit simulations were performed via CST Microwave Studio software. Scattering parameters and radiation patterns of the prototype have been verified experimentally.

## 2. Electromagnetically Coupled Microstrip Antenna

**Figure 1:**
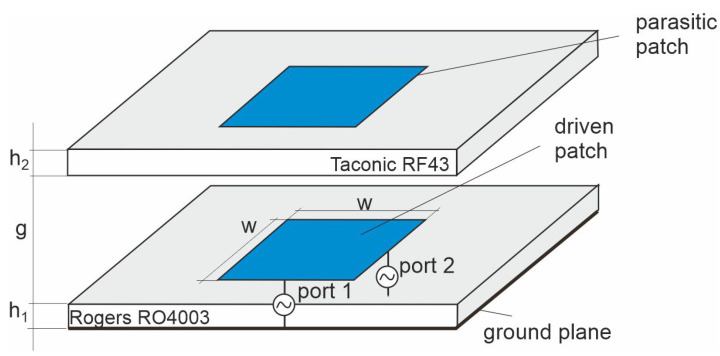
Electromagnetically coupled microstrip antennas.

The ECMS antenna utilises two substrate layers placed one above the other at a distance of g= 3 mm on which two identical square patches with a side length of w= 14 mm are located (see Figure 1). The driven patch is situated on the RO4003 substrate with a thickness of $h_{1}=$ 0.831 mm and a ground plane at the bottom. The antenna’s excitation ports locations for vertical (V) and horizontal (H) polarisations are chosen at the centres of two perpendicular sides of the patch because the application of competitive inset feed decreases port-to-port isolation. A parasitic patch is placed on the top layer of the single-sided Taconic RF43 laminate with a thickness of $h_{2}=$1.5 mm. The main goal of the parasitic patch is to widen the operational frequency band of the antenna.

**Figure 2:**
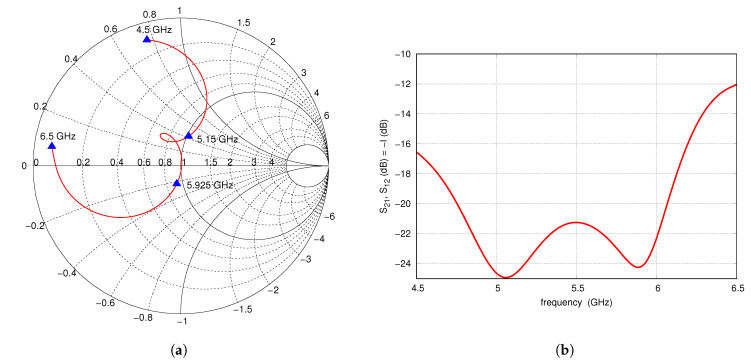
S-parameters of single ECMSA: (**a**) $s_{11}$, $s_{22}$, (**b**) $s_{21}$.

A proper selection of the type of substrates and distance between them allows the desired input impedance to be obtained for the entire assumed operational frequency band with VSWR < 1.7, which is impossible for classic microstrip antenna without a parasitic patch. The results of the full-wave analysis of S-parameters are presented in Figure 2 for a frequency ranging from 4.5 to 6.5 GHz. The limits of the antenna operating band are pointed by the markers at the Smith chart. The antenna has port-to-port isolation at a level of 21–25 dB that is a typical value for the ECMS antenna. The main lobe of the radiation pattern is normal to the patch and directivity reaches 8.35 dBi with beamwidths in the E- and H-planes of 67.3${}^{{}^{\circ}}$ and 74.3${}^{{}^{\circ}}$, respectively.

## 3. Theoretical Model

**Figure 3:**
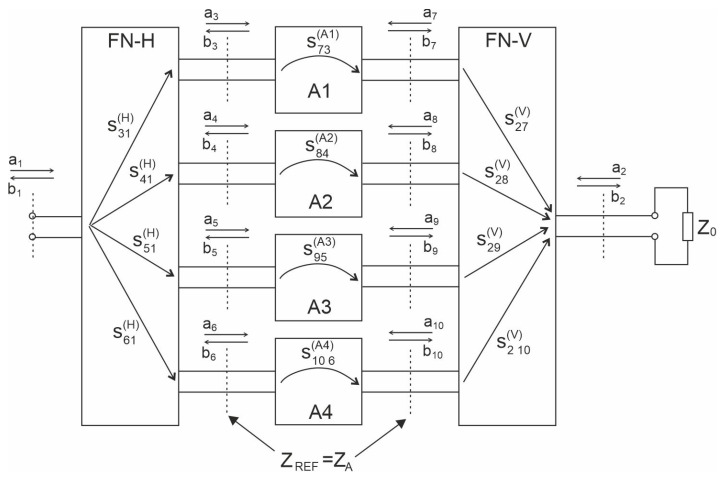
Block diagram of the array.

**Figure 4:**
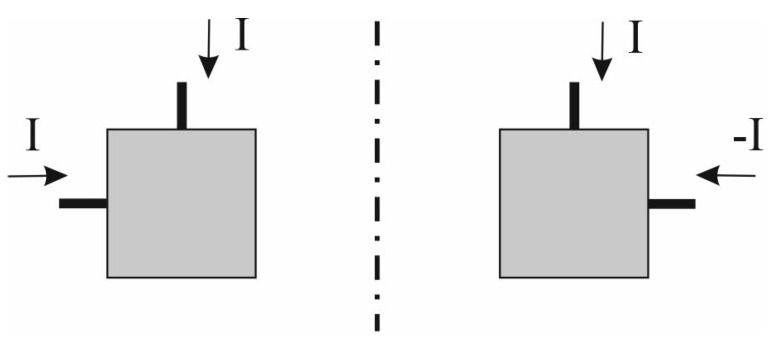
Concept of mirror imaging.

This section presents a theoretical model of port-to-port isolation for the four-element antenna array. Let us consider the operation of the four-element array built of the feeding networks for horizontal and vertical polarisations (FN-H and FN-V) and four radiators (A1 to A4) shown in Figure 3. All the included radiators are identical and form a uniform broadside array, which means that all of them are fed with currents of equal amplitudes. All the radiators are fed with the in-phase currents for horizontal polarisation, whereas for vertical polarization, the radiators could be fed with in-phase or anti-phase currents which forces the mirror imaging of the selected antennas (see Figure 4) in order to obtain the maximal radiation at the broadside direction. The reference impedance for ports 1 and 2 of the array is assumed as 50 Ω and the reference impedance of the radiator ports is equal to nominal value of their input impedance $Z_{A}$.

Port-to-port isolation of the antenna could be defined by means of the scattering parameters as

(1) $$I=\frac{a_{1}}{b_{2}}{|}_{a_{2}=0}=\frac{1}{s_{21}},$$

where $a_{1}$ denotes the incident wave and $b_{2}$ the reflected wave in ports 1 and 2, respectively. The reflection coefficients of the radiator ports are assumed to be very small ($|\Gamma_{A}|\approx 0$) which leads to the reflected waves being overlooked ($b_{3}=b_{4}=b_{5}=b_{6}\approx 0$).

The electromagnetic couplings between the adjacent antennas and/or transmission lines of the feeding network will be overlooked. The antenna ports (V and H) are assumed to be weakly coupled; therefore, the following simplifications are acceptable

$$\left|s_{73}^{\left(A1\right)}\right|\cdot \left|s_{37}^{\left(A1\right)}\right|=\left|s_{84}^{\left(A2\right)}\right|\cdot \left|s_{48}^{\left(A2\right)}\right|=\left|s_{95}^{\left(A3\right)}\right|\cdot \left|s_{59}^{\left(A3\right)}\right|=\left|s_{10\;6}^{\left(A4\right)}\right|\cdot \left|s_{6\;10}^{\left(A4\right)}\right|\ll 1,$$

which leads to the recognition that the influence of FN-H on the operating of FN-V and vice versa may be overlooked. Therefore,

(2) $$s_{21}=\frac{b_{2}}{a_{1}}{|}_{a_{2}=0}\approx \sum_{i=1}^{4}s_{(i+2)\;1}^{\left(H\right)}\cdot s_{\left(i+6)\;(i+2\right)}^{\left(Ai\right)}\cdot s_{2\;(i+6)}^{\left(V\right)}\;,$$

where $s^{\left(H\right)}$ is the transmission coefficient of the FN-H network, $s^{\left(Ai\right)}$ is the transmission coefficient of the i-th radiator and $s^{\left(V\right)}$ is the transmission coefficient of the FN-V network. In further equations, the following simplified markings are applied (i=1,…,4)

$$\begin{array}{cc} \left|s_{(i+2)\;1}^{\left(H\right)}\right|=\left|s_{1\;(i+2)}^{\left(H\right)}\right|=\left|s_{H}\right| & =\mathrm{const}., \end{array}$$

$$\begin{array}{cc} \left|s_{(i+6)\;2}^{\left(V\right)}\right|=\left|s_{2\;(i+6)}^{\left(V\right)}\right|=\left|s_{V}\right| & =\mathrm{const}.,\\ \left|s_{\left(i+6)\;(i+2\right)}^{\left(Ai\right)}\right|=\left|s_{\left(i+2)\;(i+6\right)}^{\left(Ai\right)}\right|=\left|s_{A}\right| & =\mathrm{const}. \end{array}$$

Applying all the mentioned assumptions, the incident and reflected waves as shown in the Figure 3, $s_{21}$ could be written as

(3) $$\left|s_{21}\right|=\left|s_{A}\right|\cdot \left|s_{H}\right|\cdot \left|s_{V}\right|\cdot \left|\sum_{i=7}^{10}e^{j\phi_{2i}}\right|,$$

where $\phi_{2i}$ denotes the relative phase shift angles between ports i=7,…,10 and the second port of the FN-V.

Let us consider three specific variants of the feeding network designed for a nominal frequency of $f_{0}=5.5$ GHz, which is the centre frequency of the operating frequency band. Relative phase shifts $\phi_{27}-\phi_{2\;10}$ at $f_{0}$ could have values of $\phi_{2i}=-n\cdot \pi \;\left(n=0,1,\ldots \right)$ according to the arrangement of the radiators. Additionally, when FN-V and FN-H networks are lossless, then $|s_{H}\left|=\right|s_{V}|=0.5$.

In the first variant all the radiators are fed with currents with an identical phase ($\phi_{27}=\phi_{28}=\phi_{29}=\phi_{2\;10}$), which leads to the isolation of the network equal to

(4) $$\left|s_{21}^{\left(1\right)}\right|=4\cdot \left|s_{A}\right|\cdot |s_{H}\left|\cdot \right|s_{V}|=\left|s_{A}\right|.$$

It is equal to the port-to-port isolation of a single radiator. Full compensation of the crosstalk is possible when two of the four radiators are anti-phase fed for V polarisation, i.e., $\phi_{27}=\phi_{29}$, $\phi_{28}=\phi_{2\;10}=\phi_{27}-\phi$, then (variant 2)

(5) $$\left|s_{21}^{\left(2\right)}\right|=4\cdot \left|s_{A}\right|\cdot |s_{H}\left|\cdot \right|s_{V}|\cdot \left|e^{-j\phi /2}\right|\cdot \left|\cos(\phi /2)\right|=\left|s_{A}\right|\cdot \left|\cos(\phi /2)\right|,$$

where ϕ=π at $f_{0}$. One might notice that the greater value of isolation in operating band could be obtained when the fourth radiator of the array is fed with a current with a phase shift equal to 2ϕ=2π at $f_{0}$, i.e., $\phi_{28}=\phi_{29}=\phi_{27}-\phi$ and $\phi_{2\;10}=\phi_{27}-2\phi$, then (variant 3)

(6) $$\left|s_{21}^{\left(3\right)}\right|=4\cdot \left|s_{A}\right|\cdot |s_{H}\left|\cdot \right|s_{V}|\cdot \left|e^{-j\phi }\right|\cdot \left|\cos^{2}\left(\phi /2\right)\right|=\left|s_{A}\right|\cdot \left|\cos^{2}\left(\phi /2\right)\right|.$$

**Figure 5:**
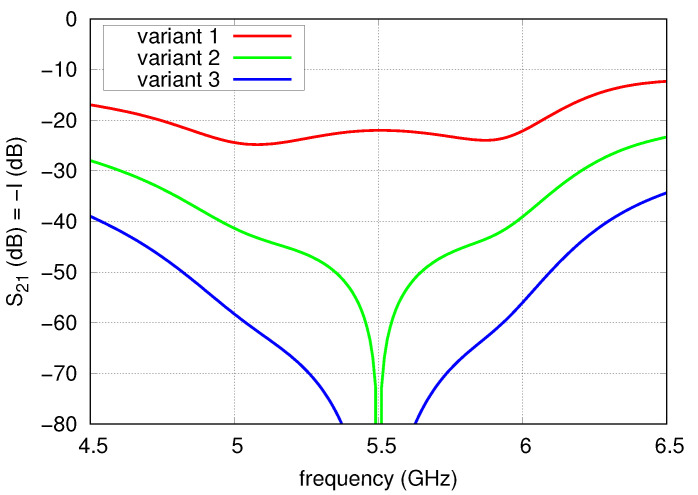
Isolation of the array for three considered variants of the feeding network.

Figure 5 presents port-to-port isolation versus frequency for three variants of the array with ECMS antennas. It was assumed that half-wave or full-wave (at $f_{0}$) sections of transmission lines were used as phase shifters. The isolation obtained in the first variant is the same as the isolation of the single radiator. In variants 2 and 3, full compensation is achieved at $f_{0}$ but in the whole U-NII operational band variant 3 features the highest isolation of 60 dB, despite the application of simple transmission line sections as phase shifters.

**Figure 6:**
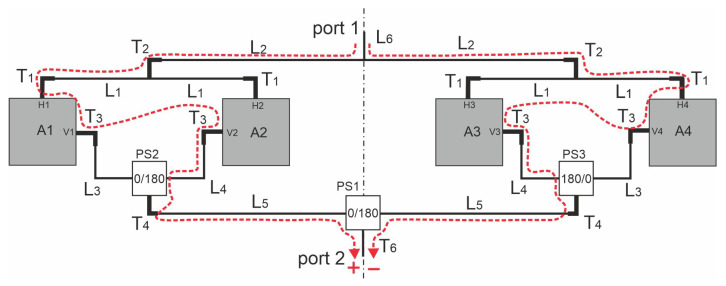
The antenna array diagram for variant 3 of the feeding network.

Practically achievable isolation is significantly influenced by electromagnetic couplings between antennas and/or microstrip lines of the FN and by the different individual values of the reflection coefficients of the radiators ports. Observed coupling levels could even be −30 dB and should be taken under consideration when designing the described antenna with an overall port-to-port isolation of 60 dB. It seems that the only successful method of minimising coupling influence is to construct an array that leads to their mutual compensation, which could be achieved by adopting mechanical symmetry within the circuit. Let us consider the possible schematic diagram for variant 3 of the feeding network of the array given in Figure 6. In the figure, the phase shifters/power dividers are denoted by PS1, PS2 and PS3. The radiators and feeding network elements maintain axial symmetry. One might observe that only in an axial symmetry circuit, the couplings omitted in the simplified circumferential analysis mutually compensate, as shown in Figure 6 for the couplings between port V1–V2 and V3–V4.

## 4. Numerical Analysis

**Figure 7:**
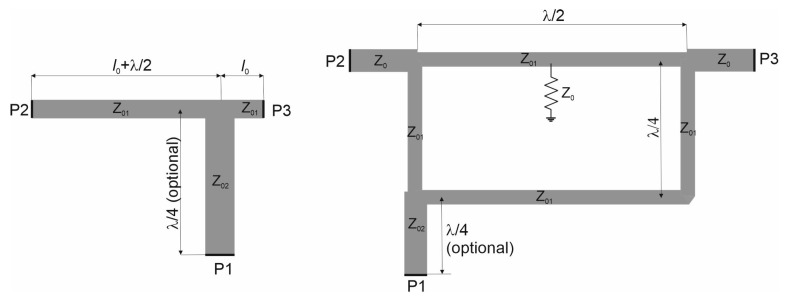
T-junction with half-wave section of transmission line (TJ) and hybrid directional coupler (HC).

**Figure 8:**
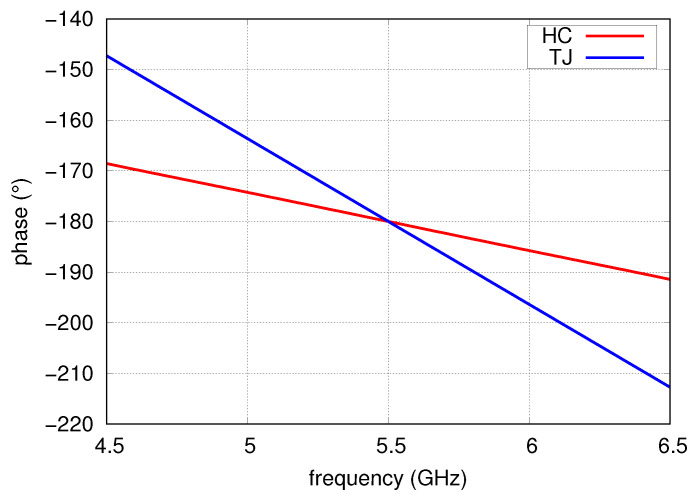
Phase shift for TJ and HC networks.

In the following section, a process of selecting the optimal arrangement of phase shifters/power dividers set by means of the circuit and full-wave simulations is described. The selection is limited to two networks: T-junction with a half-wave section of transmission line (TJ) and a hybrid directional coupler (HC) shown in Figure 7. A close look at the Formula (3) leads to the conclusion that choice of the phase shifters/power dividers should be motivated by their smaller phase variation in operating frequency band. The frequency behaviour of relative phase shift for these circuits presented in Figure 8 suggests that the optimal choice is the application of the hybrid directional couplers as PS1–PS3. An analysis of port-to-port isolation was performed considering four following possible combinations (cases) of phase shifters/power dividers:

- case A: TJ as PS1, PS2 and PS3,
- case B: HC as PS1, PS2 and PS3,
- case C: TJ as PS1 and PS2 and HC as PS3,
- case D: HC as PS1 and PS2 and TJ as PS3.

The analysis was conducted by means of circuit and full-wave simulations in order to show the influence of electromagnetic couplings between the elements of the array on the isolation obtained.

### 4.1. Analysis of the Array with the Omission of Mutual Couplings

In this case, a full-wave analysis was performed for a single separated antenna and the obtained S-parameters of the two-port network were applied as models of four elements of the antenna array in circuit simulation. Each microstrip line shown in Figure 6 was modelled as a section of an ideal transmission line whose electrical lengths at $f_{0}$ and characteristic impedances are given in Table 1.

**Figure 9:**
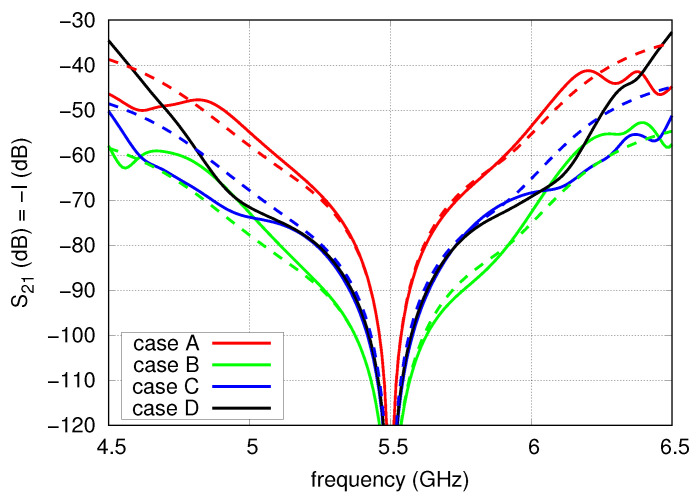
Isolation versus frequency of the four analysed cases of FN.

The simulation results are presented in Figure 9 as solid lines. Dashed lines are used to mark isolation level computed according to the derived mathematical model (3). It is visible that in the frequency band from 5.2 to 5.9 GHz, the model agrees with the characteristic achieved by the simulation when the mutual couplings between the radiators are omitted. The highest isolation close to $f_{0}$ is achieved in case B and the worst in case A. However, it should be emphasised that for an isolation level better than 60 dB, the broadest frequency band is achieved for the case C where two T-junctions and a single hybrid coupler are applied. It seems to be connected with the variations of input impedances of the antenna within the frequency band.

### 4.2. Impact of Couplings between Antennas

**Figure 10:**
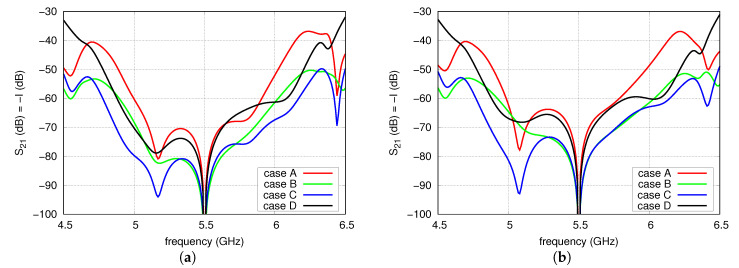
Isolation versus frequency for: (**a**) d=35 mm; (**b**) d=40 mm.

**Figure 11:**
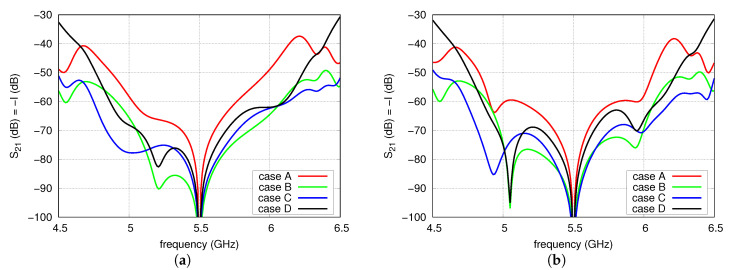
Isolation versus frequency for: (**a**) d=45 mm; (**b**) d=50 mm.

In this analysis, the couplings between the antennas were taken into account. A network built of four coupled antennas was modelled in full-wave analysis as an eight-port network. In the analysis, the excitation ports of the radiators were placed where the microstrip lines contact the patches. The calculated eight-port model, together with FN-H and FN-V models, was utilised in circuit simulations. As in the previous analysis, each microstrip line of FN-H and FN-V was modelled as a section of an ideal transmission line. As the couplings between the radiators and the isolation between their ports depend on the distance *d* between the radiators, the analysis was carried out for a number of distances from 35 to 50 mm. For each distance, electrical lengths at $f_{0}$ and impedances of the transmission lines T1–T6 and L6 were equal to the values shown in Table 1. Lengths of other lines were dependent on the distance *d* in such a way that L1 = L3 = L4 = d/2 and L2 = L5 = *d*, which correspond to the electrical lengths L1(${}^{{}^{\circ}}$) = L3 = L4 = 5.15d (mm) and L2(${}^{{}^{\circ}}$) = L5 = 10.3d (mm) of 100 Ω microstrip line on 0.831 mm thick RO4003. The results of circuit simulation are depicted in Figure 10 and Figure 11. It is observable that the maximum isolation was achieved at 5.5 GHz despite the couplings thanks to the mutual compensation effect. The impact of the couplings is observed outside of the $f_{0}$. However, it is worth noticing that the widest operational frequency band for the isolation level of 60 dB is observed in case C for all considered distances between the radiators.

### 4.3. Full-Wave Analysis

**Figure 12:**
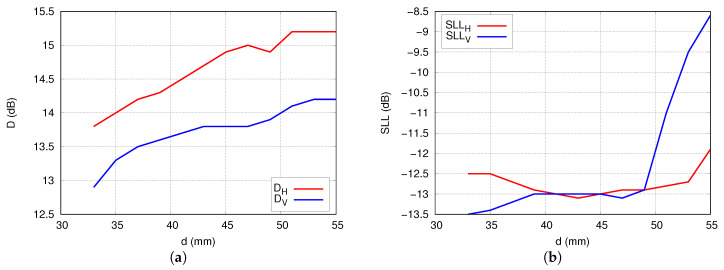
Theoretical values of the far-field parameters of the array versus distance *d* between the antennas: (**a**) directivity, (**b**) side-lobe level.

For the full-wave analysis, the combination of shifters applied in case C was selected because it offers the best chance to obtain an isolation better than the 60 dB throughout the operational frequency band. The distances between radiators were chosen to obtain the highest directivity and reasonable side lobes level (SLL). The preliminary analysis of directivity and SLL with respect to the distance *d* between the radiators was carried out applying the radiation pattern obtained as a product of radiation pattern of a single antenna and the array factor (AF). The results of the analysis are presented in Figure 12.

The directivity of the array increases with the distance *d* up to 50 mm. Unfortunately, for d> 48 mm the level of SLL for V-polarisation starts rising rapidly which is the result of grating lobes appearance. As a reasonable compromise between directivity, SLL, and the dimensions of the whole array, distance d= 45 mm was chosen.

**Figure 13:**
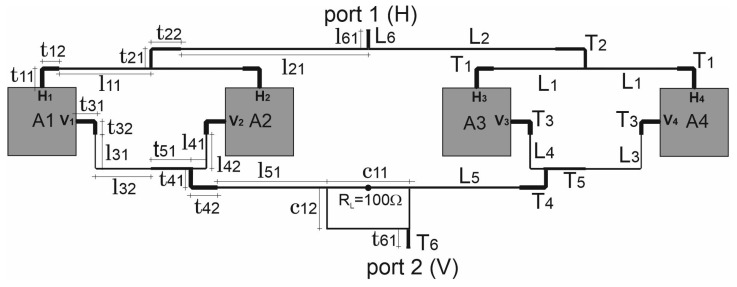
Layout of the array applied in full-wave analysis.

**Figure 14:**
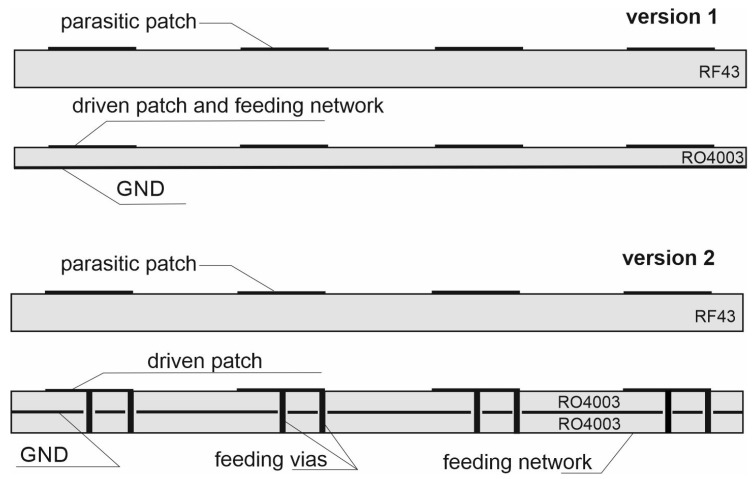
The cross-sections of the array versions.

Figure 13 shows the layout of the array which was utilised to construct a full-wave numerical model. The radiators are distinguished in gray and the feeding networks in black. The initial lengths of each transmission line and their characteristic impedances are identical as assumed in the circuit analysis. All detailed dimensions are listed in Table 2. Transformers T5 utilised in order to compensate additional attenuation caused by the variation of the lines length (applied for feeding the radiators 1 and 4) had an initial impedance of 100 Ω as L4 lines. In order to verify the influence of mutual couplings between the radiators and feeding network on port-to-port isolation, a full-wave simulation was performed for two versions of antenna construction. The cross-sections of the array versions are depicted in Figure 14. In version 1, the radiators and the feeding network are placed on the same layer. In version 2, the radiators and the feeding network are separated by the ground plane.

The full-wave simulations of versions 1 and 2 demonstrated that the minimal isolation in the whole operating band is 51.6 and 59.8 dB, respectively. Anticipating an improvement in the parameters of the antenna array versions, an optimisation of their FNs was performed in order to take into account the influence of the applied technology elements (application of vias, coaxial connectors) and couplings between the sections of transmission lines. For instance, considering the vias led to the modification of characteristic impedances and electrical lengths of T1 and T3 transformers. For both versions, a manual optimisation process was performed basing on the knowledge and experience of the authors. In fact, this involved a set of full-wave analyses made with small variations in the impedance and length of the chosen transmission lines. Additionally, it was assumed that the optimisation should not cause an increase of $s_{11}$ and $s_{22}$ over −9.54 dB (VSWR < 2) within the whole operational band.

Optimised dimensions and characteristic impedances of the transmission lines for both versions are given in Table 2. Additionally, it was discovered for both versions that omitting the loading resistor of the hybrid coupler causes an isolation increment without significantly affecting the matching of the network, which further simplifies the design of the arrays.

**Figure 15:**
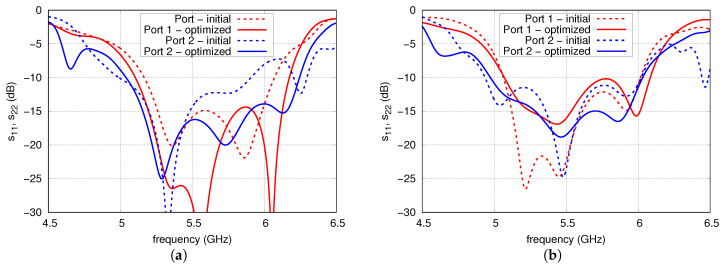
The $s_{11}$ and $s_{22}$ of initial and optimised array for: (**a**) version 1; (**b**) version 2.

**Figure 16:**
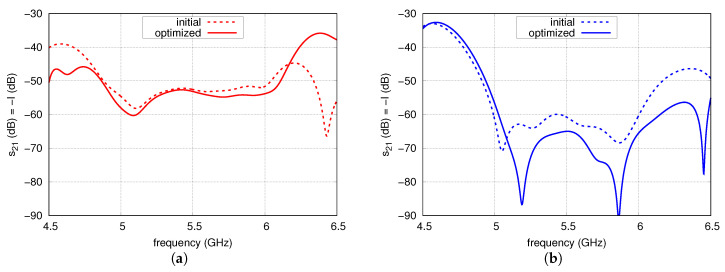
The $s_{21}$ of initial and optimised array for: (**a**) version 1; (**b**) version 2.

The $s_{11}$ and $s_{22}$ of versions 1 and 2 before and after the optimisation are shown in Figure 15. The result of optimisation performed for version 1 is widening of the impedance bandwidth for both ports and lowering the $s_{22}$. For version 2, the result is widening of the impedance bandwidth at the expense of increasing $s_{11}$ to −10 dB and lowering the maximal $s_{22}$ together with slight narrowing the impedance band. The $s_{21}$ before and after the optimisation is depicted in Figure 16. In the case of version 1, the isolation has been improved to just an average of 1.2 dB. A greater improvement was achieved for version 2 where the minimal increment of the isolation is 3 dB and its mean value is 8.5 dB for the whole U-NII frequency band.

Analysis of the results shows that it is possible to achieve isolation to the level of 50 dB for the single-layer topology, which is significantly worse than in circuit simulation due to strong couplings between the radiators and FN. Weaker couplings for the multi-layer topology results in an isolation better than 65 dB in U-NII frequency band, which is on average 6.8 dB less than in circuit simulations.

## 5. Experimental Verification

**Figure 17:**
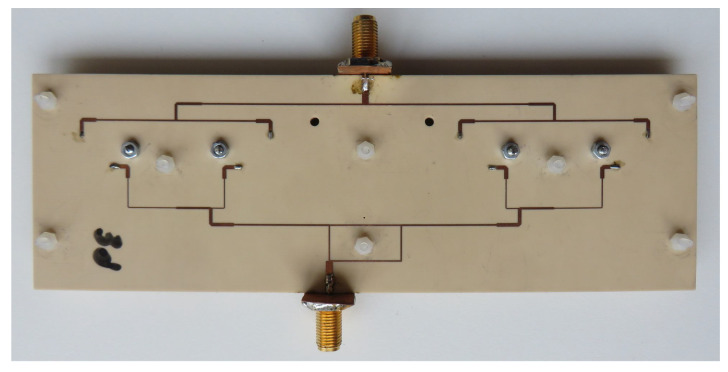
Fabricated antenna array—bottom view.

**Figure 18:**
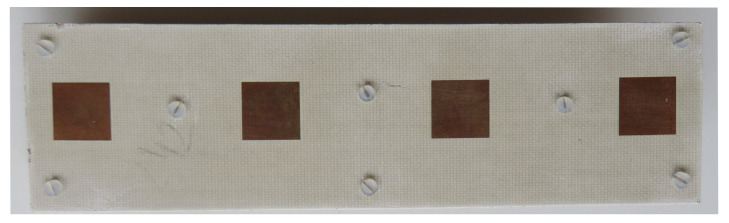
Fabricated antenna array—top view.

In this section, the results of S-parameters and radiation patterns obtained by means of measurements are compared to the full-wave simulation results. The antenna prototype used was version 2, which has much better isolation in comparison to version 1. The photographs of the antenna assembled by the authors are presented in Figure 17 and Figure 18.

**Figure 19:**
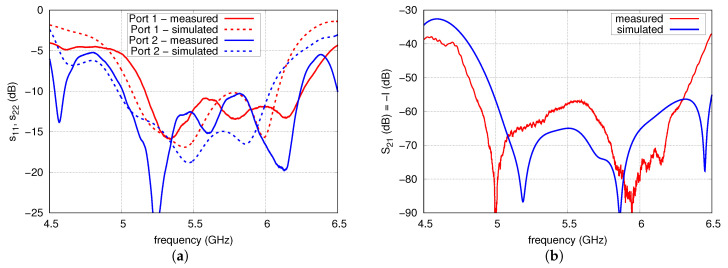
Measured and simulated S-parameters of the antenna array: (**a**) $s_{11}$, $s_{22}$, (**b**) $s_{21}$.

Circuit parameters of the array have been measured with the vector network analyser (N5230A, Agilent Technologies, Malaysia) in a frequency range from 4.5 to 6.5 GHz. A comparison of the results of S-parameters obtained with simulation and measurements is presented in Figure 19. As can be observed, a good agreement is achieved, although the measured operational frequency band of the antenna is slightly shifted towards the higher frequencies compared to the results of the simulations. The VSWR < 2 ($s_{11}$ and $s_{22}$ less than −9.54 dB) is achieved from 5.14 to 6.26 GHz for port 1 and from 5 to 6.24 GHz for port 2. In the majority of the antenna frequency band, the isolation is better than 60 dB. Only from 5.39 to 5.67 GHz is the isolation slightly smaller and reaches a minimum value of 57 dB. Comparing the results obtained by simulation and measurements, it can be observed that in the operational frequency band, the characteristics of antenna isolation are very similar, although the measurements results are about 10 dB worse than in the simulations. According to the authors, it would be possible to achieve a better agreement of the results if the antenna were assembled professionally.

**Figure 20:**
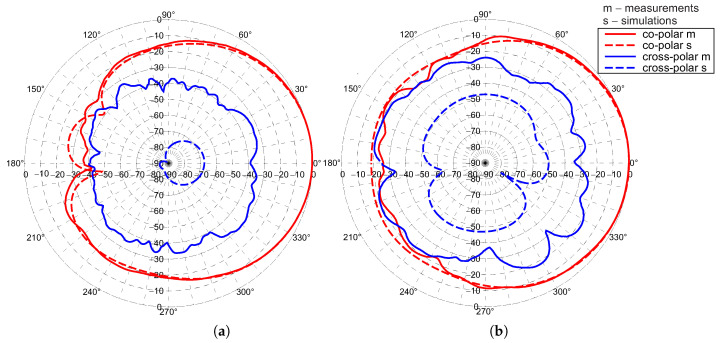
Normalised co-polar and cross-polar radiation patterns measured and simulated at 5.5 GHz on the principal H-plane for: (**a**) port 1; (**b**) port 2.

**Figure 21:**
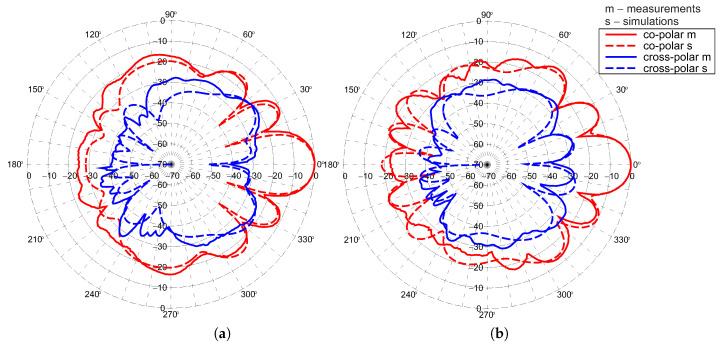
Normalised co-polar and cross-polar radiation patterns measured and simulated at 5.5 GHz on principal V-plane for: (**a**) port 1; (**b**) port 2.

Radiation patterns of the antenna array were measured in an anechoic chamber at 5.5 GHz. The co-polar and cross-polar patterns in H and V planes for port 1 and 2 are presented in the Figure 20 and Figure 21, respectively. Good agreement of simulation and measurements results in the case of co-polar patterns is observed. For cross-polar patterns in the V-plane the agreement can be also considered as satisfactory. Significant differences in simulation and measurements for cross-polar pattern in H-plane result from the fact that in this direction the simulated level of radiation approaches zero. The gain, beamwidth and SLL are almost steady in the operational frequency band. The gains are equal to 14.3 and 13.2 dBi for port 1 and port 2, respectively. The lower level of the gain for port 2 is the result of the wider main lobe in H-plane. The SLL in V-plane is −11.9 dB for port 1 and −10.5 dB for port 2. Antenna has very good polarisation purity for broadside direction. The array has a cross-polarisation level close to −27 dB within the main lobe, which should be considered as a good result.

In Table 3, the prototype array is compared with other dual-polarised linear arrays described in the literature. Only in [15] is the pairwise antiphase feeding technique applied. In all other mentioned papers, the radiators are fed in-phase for both polarisations. In [15], despite the application of the pairwise antiphase feeding technique, the result is only a slight improvement of isolation to 36 dB. This is the result of a non-optimal choice of phase shifts for currents feeding the individual radiators. In [23,32,33] the isolation of the array is 25–31 dB, which is similar to the isolation of the applied single antenna. In [29,30] the isolation of a single radiator is relatively high (38–39 dB) due to differential feeding. Despite this, the isolation of the array is significantly worse (30 dB). This seems to be the effect of couplings between array elements. In [25], isolation of 50 dB is obtained in a narrow frequency band thanks to the use of a directional coupler separating networks for both polarisations, despite the use of FN that do not improve the isolation. In the proposed antenna array, the isolation is 7–32 dB better than the competitor arrays thanks to the use of a carefully chosen and optimised feeding network. The high isolation was obtained despite the application of low isolation radiators. The gain is similar to other four-element competitors and the cross polarisation is comparable to the best results of the listed arrays.

## 6. Conclusions

The article describes in detail the designing of dual-polarised linear antenna arrays with increased port-to-port isolation based on the example of a four-element array dedicated to the 5.15–5.925 GHz U-NII band. Despite the use of electromagnetically coupled microstrip antennas featuring isolation less than 25 dB, the designed array is characterised by isolation better than 65 dB in the operating band according to the results of full-wave simulations. This was achieved thanks to the proper design of the feeding network compensating crosstalk between the ports. The optimal feeding network was proposed based on a derived theoretical model describing the isolation of the antenna array. An optimal configuration of power dividers/phase shifters was chosen by means of the circuit and full-wave simulations. It should be emphasised that selection of the feeding network with larger relative phase shift fluctuations in the operating band is a more advantageous variant of the proposed antenna version. The prototype of the array was verified experimentally by the scattering parameters and the radiation pattern measurements. It was demonstrated that in the operational band, the antenna prototype is characterised by a gain of about 14 dBi, isolation better than 57 dB and polarisation purity of at least −27 dB within the main lobe.

## Figures and Tables

**Table 1:** Electrical lengths and characteristic impedances of the applied ideal transmission lines.

	T1	T2	T3	T4	T6	L1	L2	L3	L4	L5	L6
L (${}^{{}^{\circ}}$)	71	90	71	90	90	205.2	428.4	205.2	205.2	428.4	90
$Z_{0}\left(\Omega \right)$	64	70.7	64	70.7	50	100	100	100	100	100	50

**Table 2:** Initial (I) and optimised (O) parameters of FN microstrip lines for version 1 and version 2.

		Version 1						Version 2					
		*l* (mm)		*w* (mm)		$Z_{0}$ (Ω)		*l* (mm)		*w* (mm)		$Z_{0}$ (Ω)	
		I	O	I	O	I	O	I	O	I	O	I	O
T1	$t_{11}$	3.225	4	1.17	0.96	64	70.7	3.225	4	1.17	0.96	64	70.7
	$t_{12}$	3.225	3.5	1.17	0.96	64	70.7	3.225	3.5	1.17	0.96	64	70.7
T2	$t_{21}$	4.165	4	0.96	0.7	70.7	82.5	4.165	4	0.96	0.7	70.7	82.5
	$t_{22}$	4.165	6.2	0.96	0.7	70.7	82.5	4.165	6.2	0.96	0.7	70.7	82.5
T3	$t_{31}$	3.255	4	1.17	1	64	69.6	3.255	4	1.17	1	64	69.6
	$t_{32}$	3.225	4.25	1.17	1	64	69.6	3.225	2.75	1.17	1	64	69.6
T4	$t_{41}$	4.165	3.9	0.96	0.96	70.7	70.7	4.165	3.9	0.96	0.85	70.7	75.4
	$t_{42}$	4.165	5	0.96	0.96	70.7	70.7	4.165	5.5	0.96	0.85	70.7	75.4
T5	$t_{51}$	8.54	8.25	0.46	0.55	98	91.4	8.54	8.25	0.46	0.55	98	91.4
T6	$t_{61}$	–	–	–	–	–	–	8.33	7.8	0.96	1.7	70.7	52.1
L1	$l_{11}$	19.5	19	0.435	0.44	100	99.6	19.5	19	0.435	0.44	100	99.6
L2	$l_{21}$	40.7	38.8	0.435	0.55	100	91.4	40.7	38.8	0.435	0.44	100	99.6
L3	$l_{31}$	7.25	7	0.435	0.27	100	117.7	7.25	7	0.435	0.27	100	117.7
	$l_{32}$	12.26	11.5	0.435	0.27	100	117.7	12.26	11.5	0.435	0.27	100	117.7
L4	$l_{41}$	3.7	3.25	0.435	0.27	100	117.7	3.7	3.25	0.435	0.27	100	117.7
	$l_{42}$	7.25	7	0.435	0.27	100	117.7	7.25	7	0.435	0.27	100	117.7
L5	$l_{51}$	23.8	23.2	0.435	0.44	100	99.6	23.8	22.7	0.435	0.44	100	99.6
HC	$c_{11}$	17.52	17.08	0.14	0.35	141	108.1	17.52	17.08	0.14	0.25	141	120.5
	$c_{12}$	8.76	8.54	0.14	0.35	141	108.1	8.76	8.54	0.14	0.25	141	120.5
L6	$l_{61}$	–	–	–	–	–	–	8.12	5	1.8	1.6	50	53.7

*l*—line length, *w*—line width, $Z_{0}$—line impedance.

**Table 3:** Comparison of the proposed and reference antennas.

Reference	Number of Elements	Bandwidth (GHz)	I of Single Antenna (dB)	I of Antenna Array (dB)	Gain (dBi)	Cross-Pola- Rization (dB)
[15]	4	8.25–9.5 (14%)	–	>34	–	<−27
[23]	6	1.71–2.69 (44%)	>30	>31	15 ± 1.1	<−11.5
[25]	4	4.75–5.18 (9%)	–	>50	>11	<24
[29]	4	1.65–2.72 (49%)	>38	>30	13 ± 0.8	<−10
[30]	4	1.66–2.93 (53.9%)	>39	>30	13.2 ± 0.7	<−18
[31]	4	1.55–2.5 (47%)	>30	>36	13.7 ± 0.2	<−27
[32]	4	2.4–2.65 (10%)	>30	>30	12.5 ± 1.5	<−18.7
[33]	6	1.8–4 (75.9%)	>25	>25	15.6 ± 1.2	<−20
this work	4	5.15–5.925 (20%)	>25	>57	13.8 ± 0.5	<−27

## Abbreviations

The following abbreviations are used in this manuscript:

U-NII	Unlicensed National Information Infrastructure
MIMO	Multiple Input Multiple Output
SCFD	Single-Channel Full Duplex
STAR	Simultaneous Transmit and Receive
FN	Feeding Network
PCB	Printed Circuit Board
ECMSA	Electromagnetically Coupled Microstrip Antenna
VSWR	Voltage Standing Wave Ratio
SLL	Side Lobe Level
AF	Array Factor
